# Supplementary material for: Composition Characterization of Cinnamomum osmophloeum Kanehira Hydrosol and Its Enhanced Effects on Erectile Function
Source: Plants (Basel). 2024 May 31;13(11):1518. doi: 10.3390/plants13111518 (PMC11174926; doi:10.3390/plants13111518)
Supplement: Supplementary file 1 [file plants-13-01518-s001.zip › plants-3021885-supplementary.pdf]

Supporting Information

Composition Characterization of *Cinnamomum osmophloeum* Kanehira Hydrosol and Its Enhanced Effects on Erectile Function

Chung-Hsuan Wang [1,2], Nai-Wen Taso [1], Chi-Jung Chen [3], Hung-Yi Chang [3] and Sheng-Yang Wang [1,2,4,*]

1 Special Crop and Metabolome Discipline Cluster, Academy Circle Economy, National Chung Hsing University, Taichung 402202, Taiwan; piscium0312@smail.nchu.edu.tw (C.-H.W.); nwt1228@dragon.nchu.edu.tw (N.-W.T.)

2 Department of Forestry, National Chung Hsing University, Taichung 402202, Taiwan

3 Taichung Branch, Forestry and Nature Conservation Agency, Ministry of Agriculture, Taichung 402212, Taiwan; chenchijung@gmail.com (C.-J.C.); changgingo@gmail.com (H.-Y.C.)

4 Agricultural Biotechnology Research Center, Academia Sinica, Taipei City 115201, Taiwan

* Correspondence: taiwanfir@dragon.nchu.edu.tw; Tel.: +886-422-850-333; Fax: +886-422-862-960

Figure S1. ^{1}H NMR spectrum of *trans*-methyloxetan-3-ol (**1**).. 3

Figure S2. ^{13}C NMR spectrum of *trans*-methyloxetan-3-ol (**1**).. 3

Figure S3. ^{1}H NMR spectrum of *cis*-methyloxetan-3-ol (**2**). 4

Figure S4. ^{13}C NMR spectrum of *cis*-methyloxetan-3-ol (**2**). 4

Figure S5. ^{1}H NMR spectrum of 3-methoxy-4-hydroxyphenylglycol (**3**)................................ 5

Figure S6. ^{13}C NMR spectrum of 3-methoxy-4-hydroxyphenylglycol (**3**). 5

Figure S7. ^{1}H NMR spectrum of *cis*-phenyloxetan-3-ol (**4**)... 6

Figure S8. ^{13}C NMR spectrum of *cis*-phenyloxetan-3-ol (**4**).. 6

Figure S9. ^{1}H NMR spectrum of 5-(2-hydroxypropan-2-yl)-2-methylcyclohex-3-ene-1,2-diol (**5**). 7

Figure S10. ^{13}C NMR spectrum of 5-(2-hydroxypropan-2-yl)-2-methylcyclohex-3-ene-1,2-diol (**5**). 7

Figure S11. ^{1}H NMR spectrum of *trans*-phenyloxetan-3-ol (**6**)... 8

Figure S12. ^{13}C NMR spectrum of *trans*-phenyloxetan-3-ol (**6**).. 8

Figure S13. ^{1}H NMR spectrum of 2-(2-hydroxyphenyl)oxetan-3-ol (**7**)................................. 9

Figure S14. ^{13}C NMR spectrum of 2-(2-hydroxyphenyl)oxetan-3-ol (**7**)................................. 9

Figure S15. DEPT spectrum 2-(2-hydroxyphenyl)oxetan-3-ol (**7**)... 10

Figure S16. HSQC spectrum of 2-(2-hydroxyphenyl)oxetan-3-ol (**7**). 10

Figure S17. HMBC spectrum of 2-(2-hydroxyphenyl)oxetan-3-ol (**7**). 11

Figure S18. COSY spectrum of 2-(2-hydroxyphenyl)oxetan-3-ol (**7**). 11

Figure S19. NOESY spectrum of 2-(2-hydroxyphenyl)oxetan-3-ol (**7**)................................. 12

Figure S20. Cytotoxicity of CO hydrosol and its compounds on Hep3B 12

Figure S1. ^{1}H NMR spectrum of *trans*-methyloxetan-3-ol (**1**).

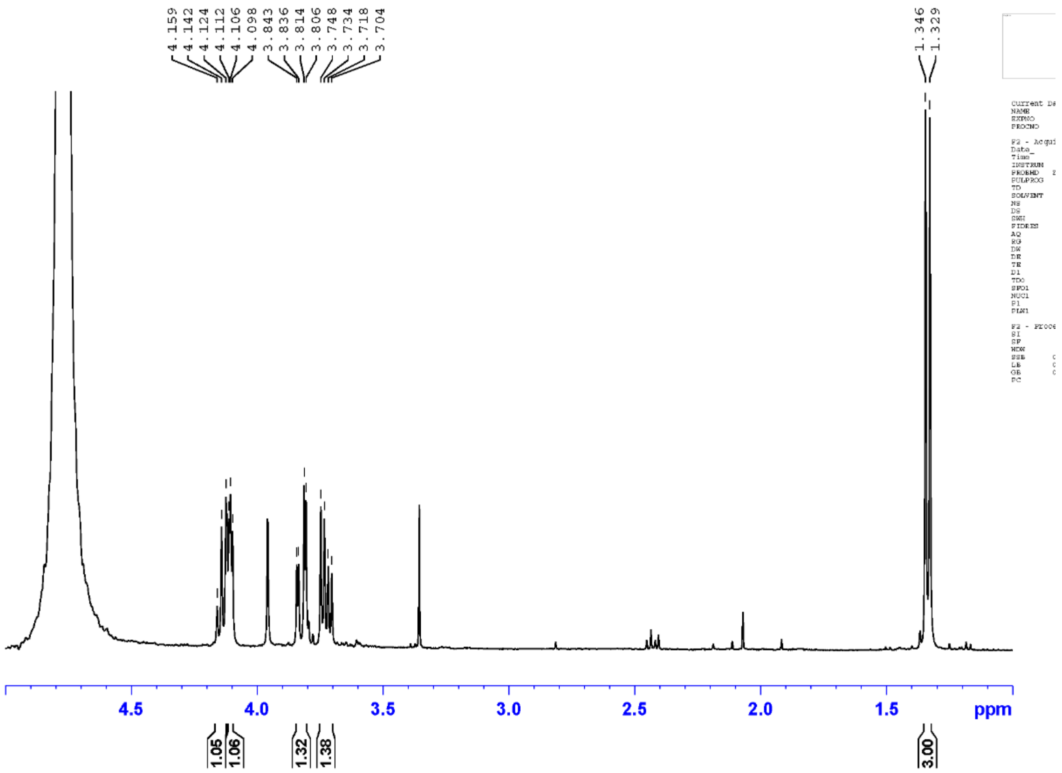

Figure S2. ^{13}C NMR spectrum of *trans*-methyloxetan-3-ol (**1**).

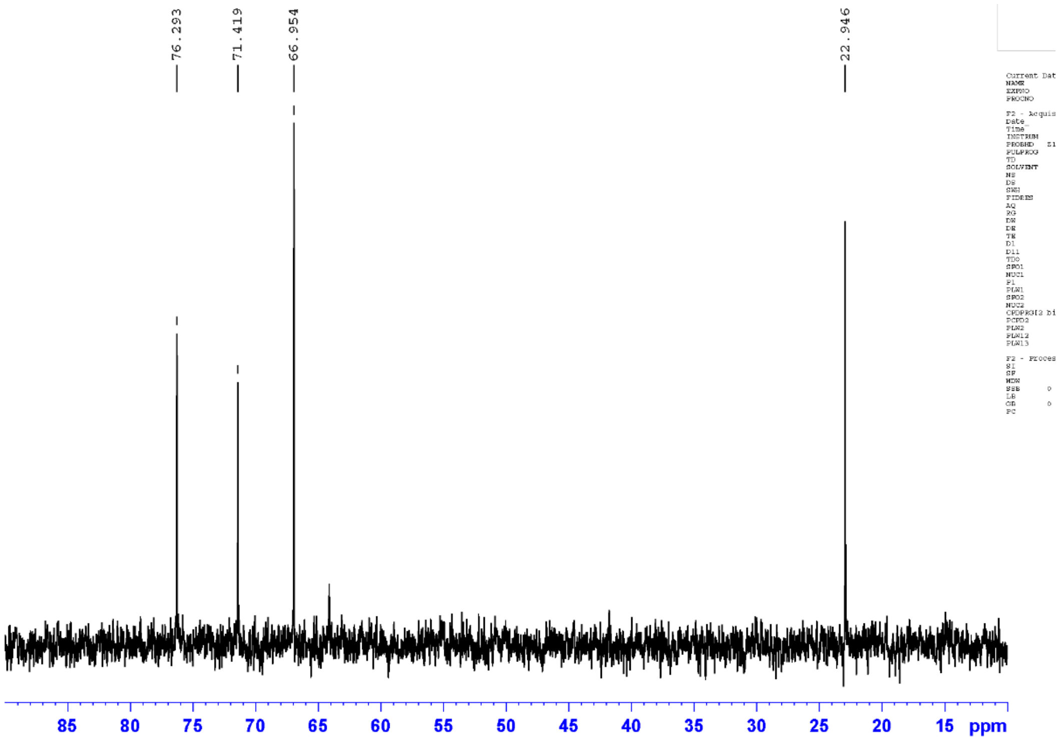

Figure S3. ^{1}H NMR spectrum of *cis*-methyloxetan-3-ol (**2**).

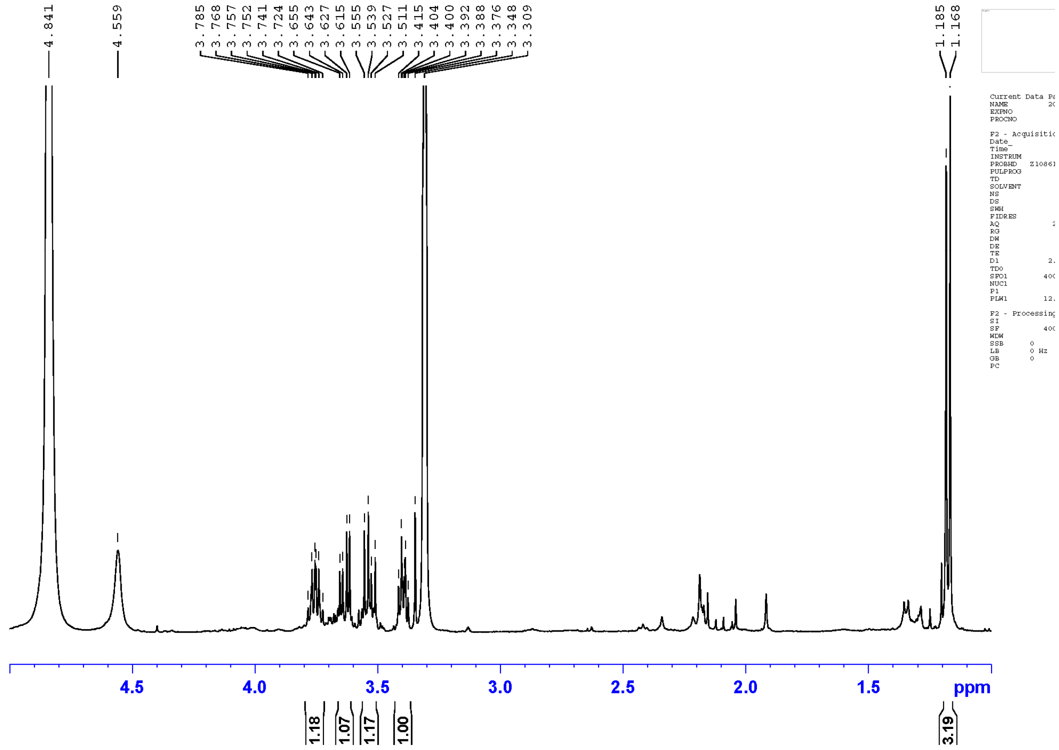

Figure S4. ^{13}C NMR spectrum of *cis*-methyloxetan-3-ol (**2**).

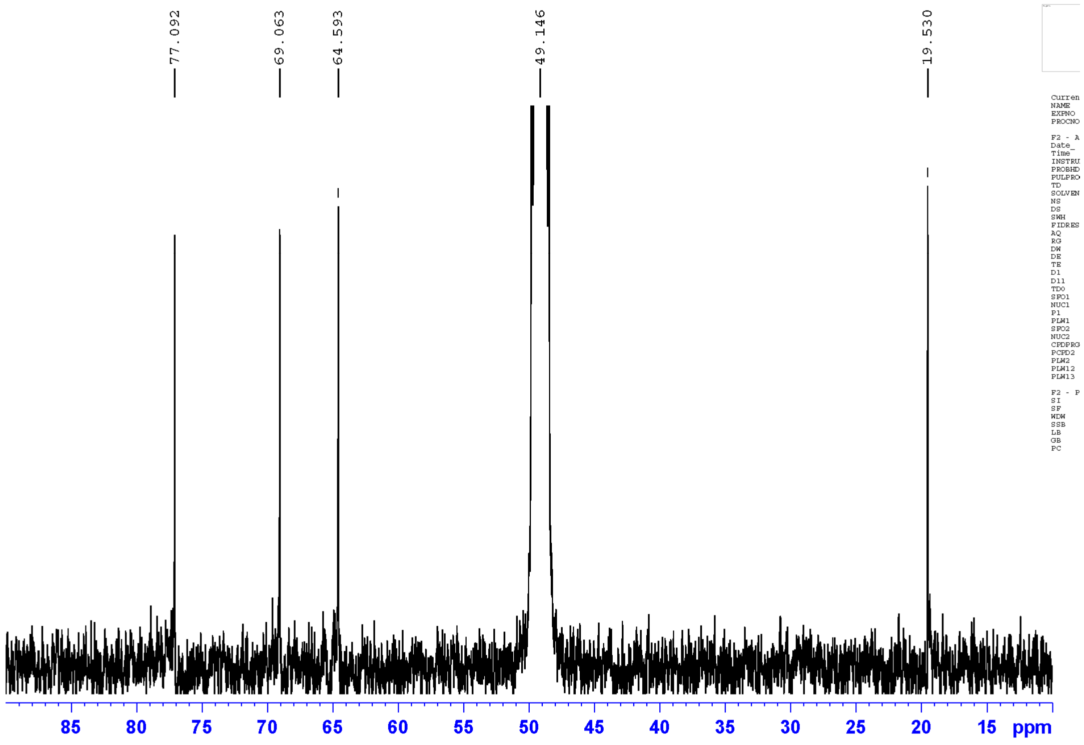

Figure S5. ^{1}H NMR spectrum of 3-methoxy-4-hydroxyphenylglycol (**3**).

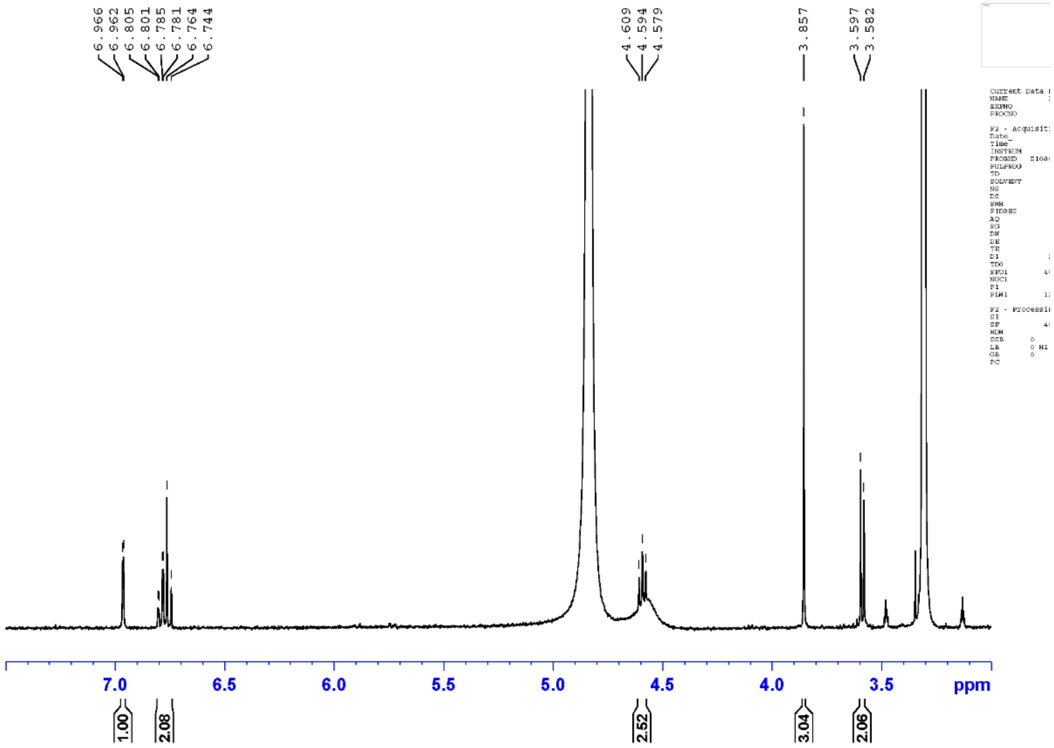

Figure S6. ^{13}C NMR spectrum of 3-methoxy-4-hydroxyphenylglycol (**3**).

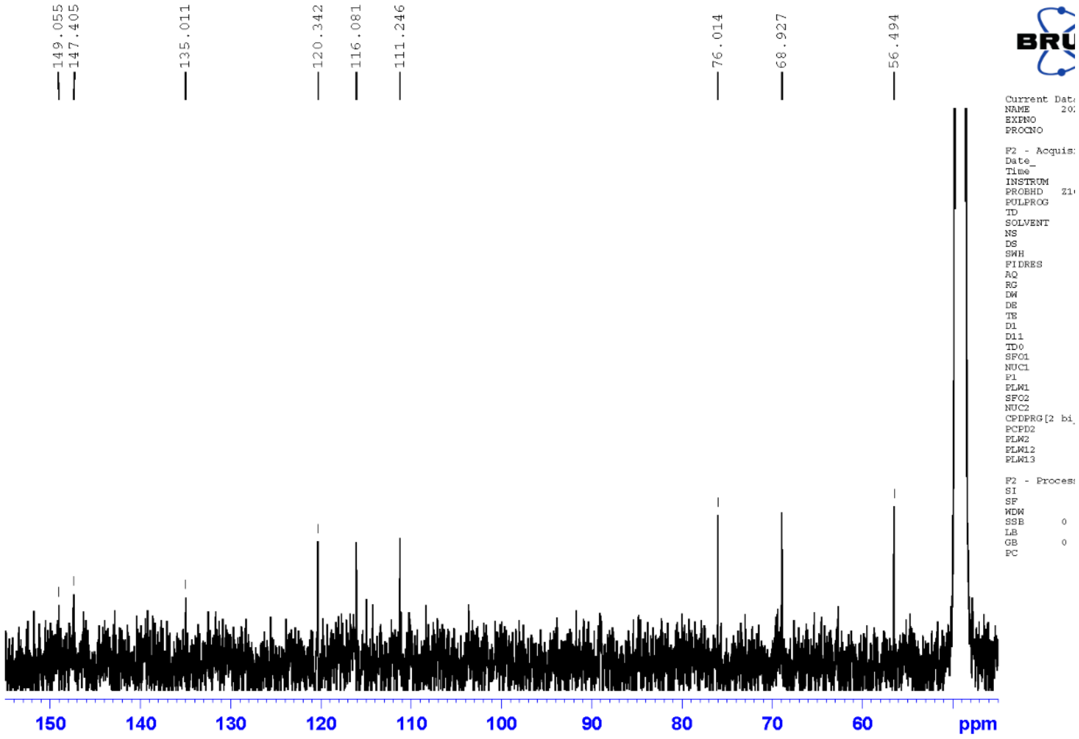

Figure S7. ^{1}H NMR spectrum of *cis*-phenyloxetan-3-ol (**4**).

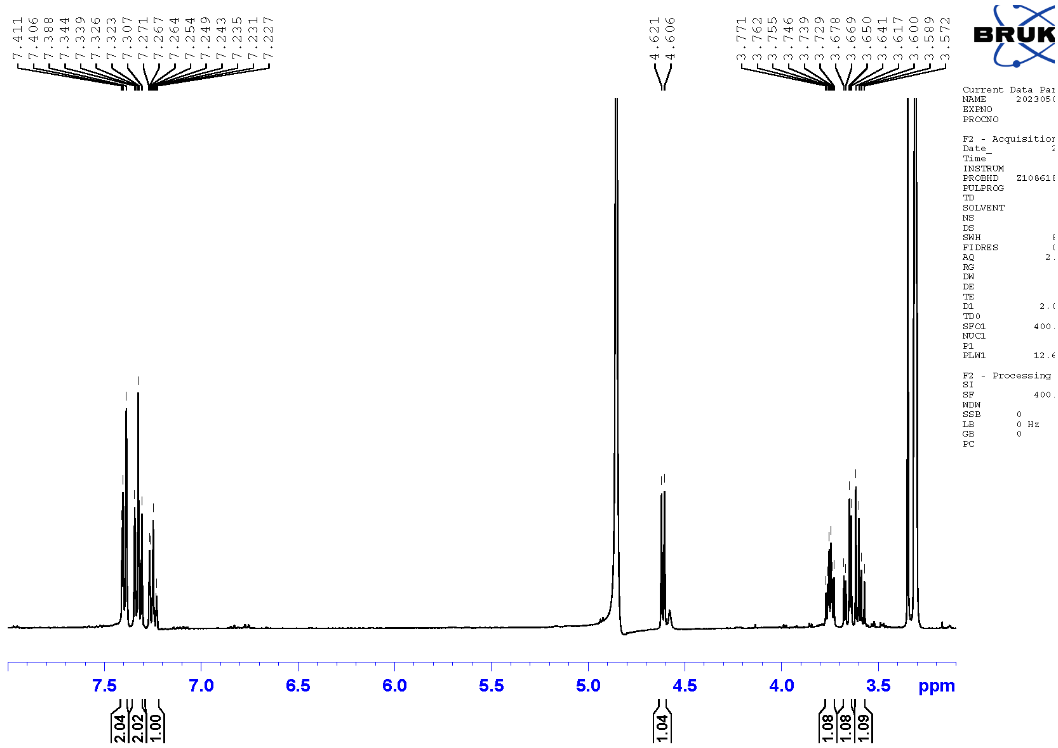

Figure S8. ^{13}C NMR spectrum of *cis*-phenyloxetan-3-ol (**4**).

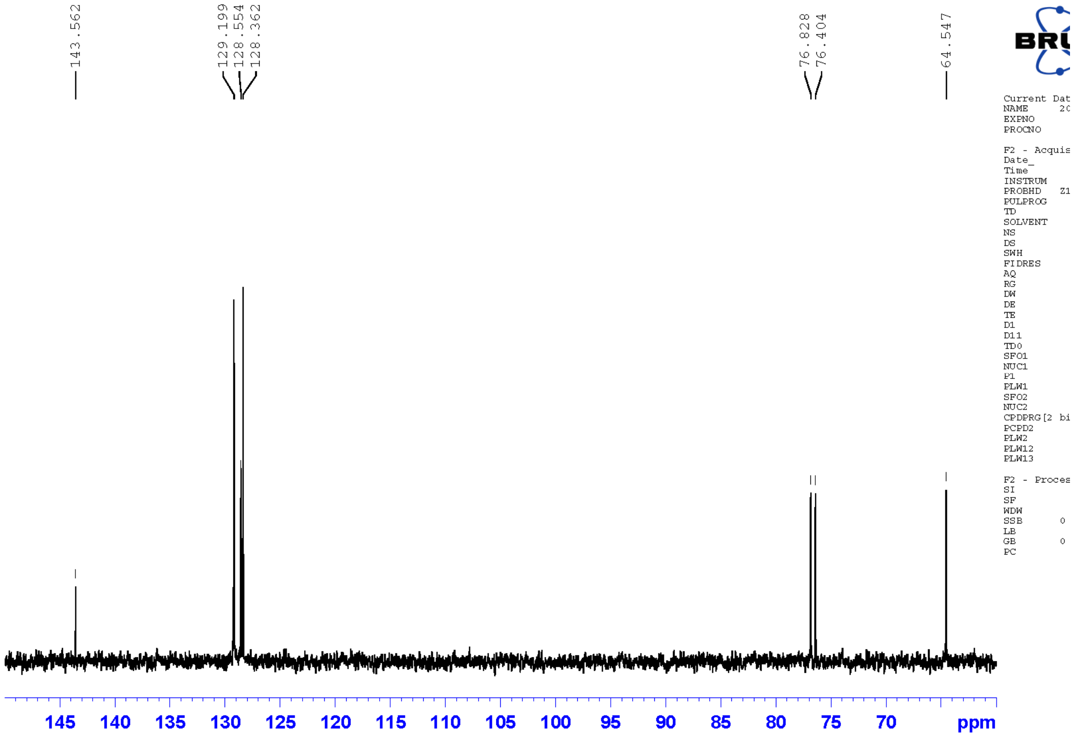

Figure S9. ^{1}H NMR spectrum of 5-(2-hydroxypropan-2-yl)-2-methylcyclohex-3-ene-1,2-diol (**5**).

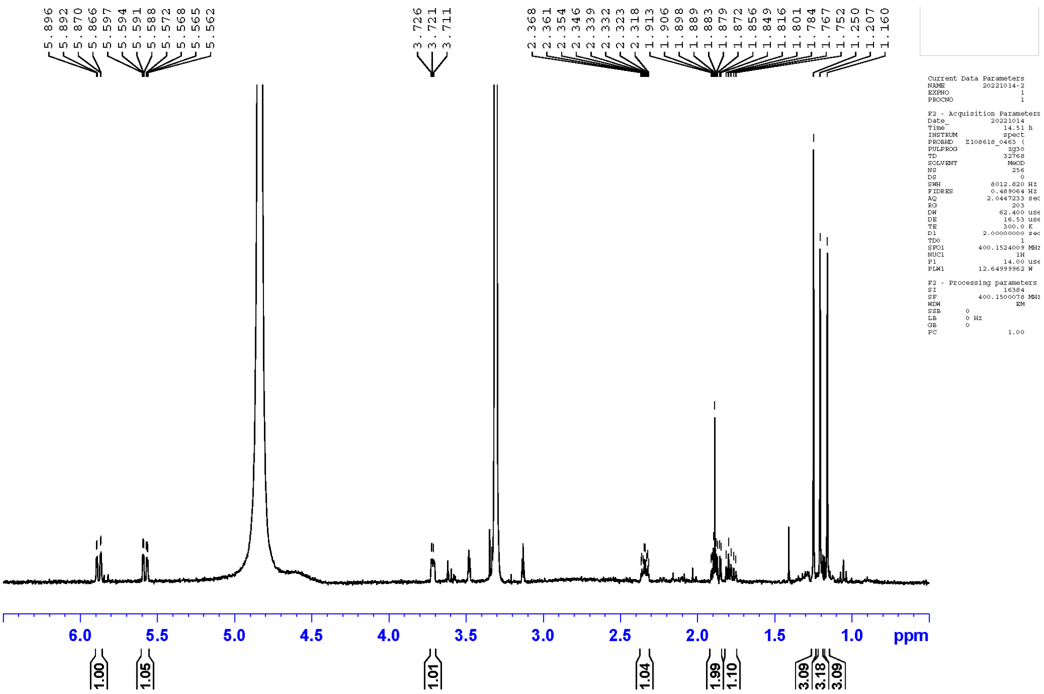

Figure S10. ^{13}C NMR spectrum of 5-(2-hydroxypropan-2-yl)-2-methylcyclohex-3-ene-1,2-diol (**5**).

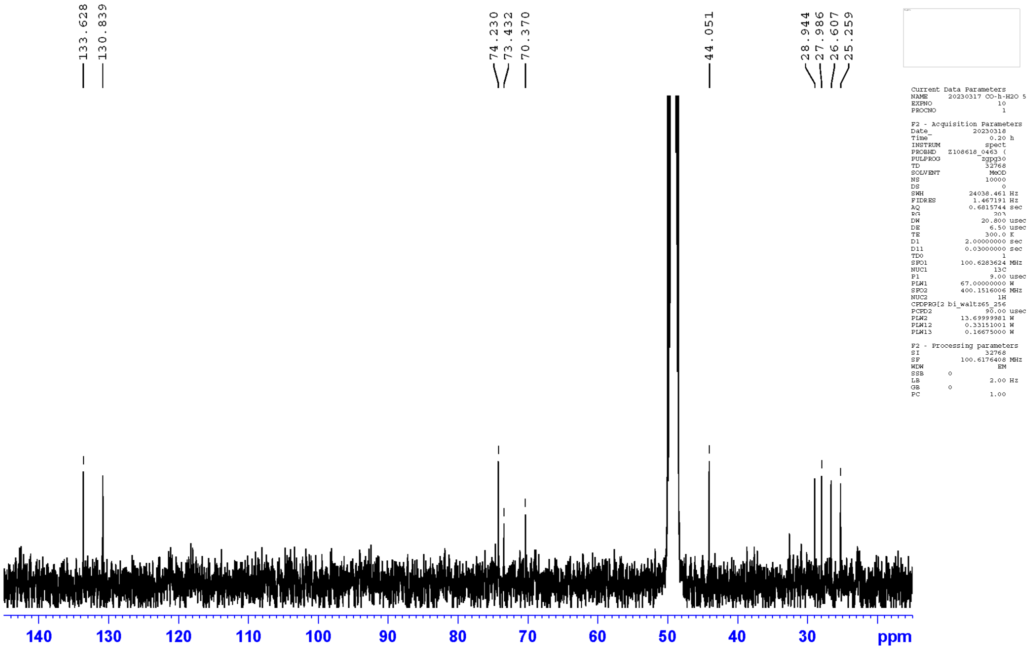

Figure S11. ^{1}H NMR spectrum of *trans*-phenyloxetan-3-ol (**6**).

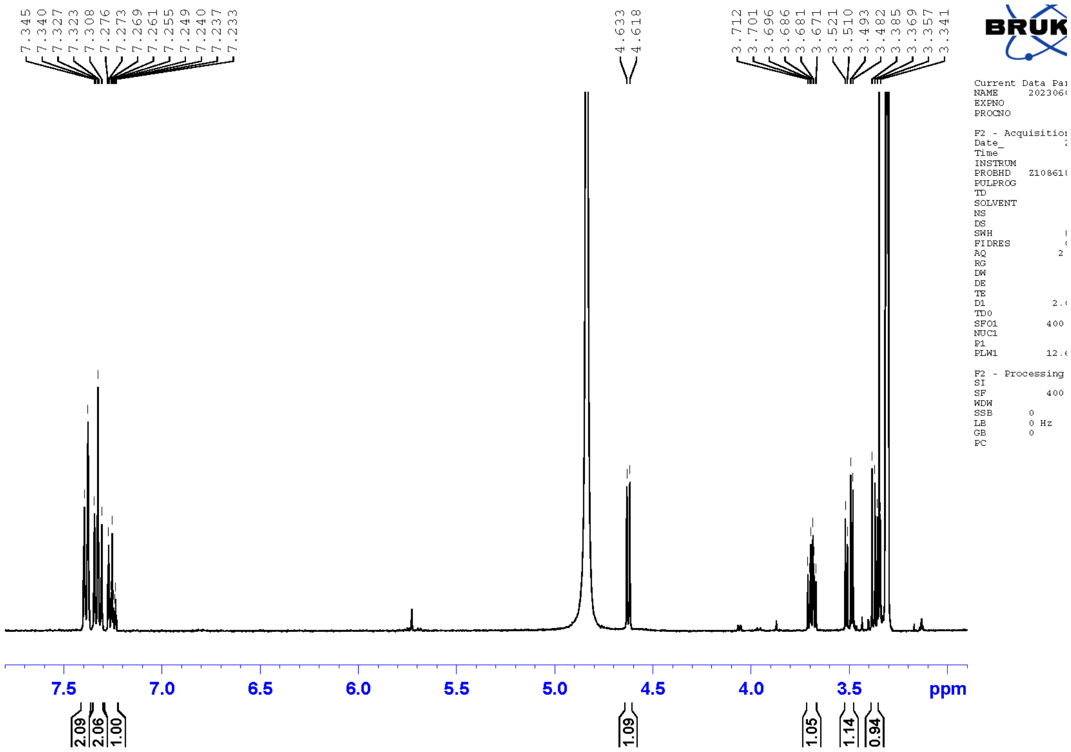

Figure S12. ^{13}C NMR spectrum of *trans*-phenyloxetan-3-ol (**6**).

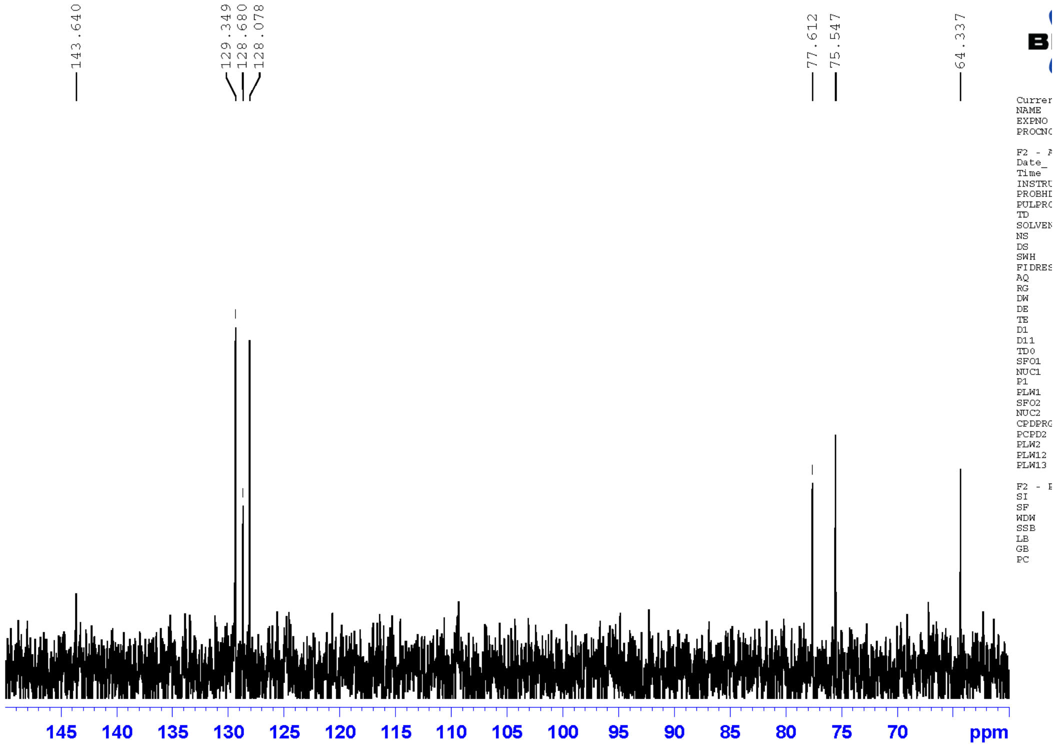

Figure S13. ^{1}H NMR spectrum of 2-(2-hydroxyphenyl)oxetan-3-ol (**7**).

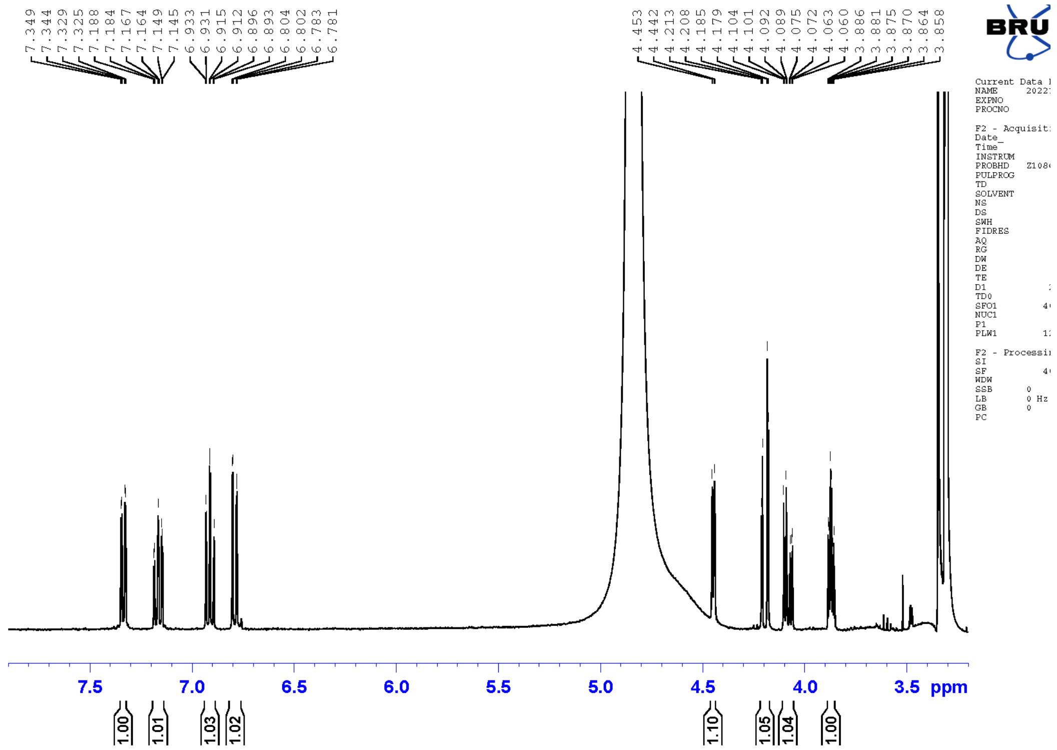

Figure S14. ^{13}C NMR spectrum of 2-(2-hydroxyphenyl)oxetan-3-ol (**7**).

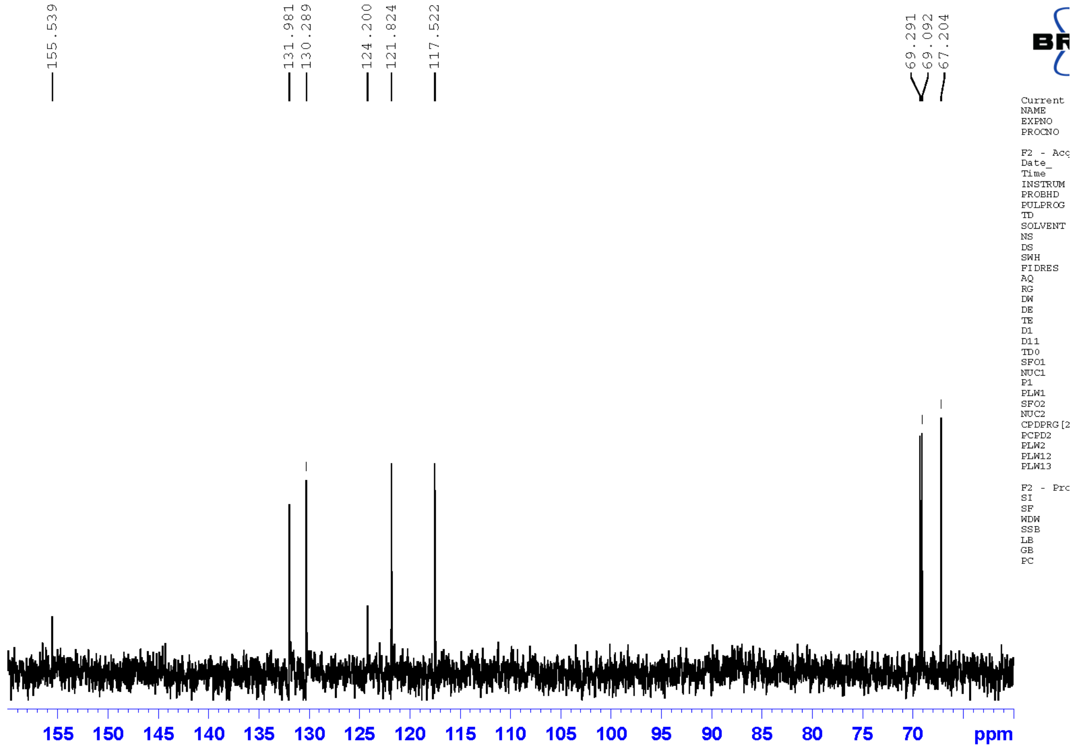

Figure S15. DEPT spectrum 2-(2-hydroxyphenyl)oxetan-3-ol (**7**).

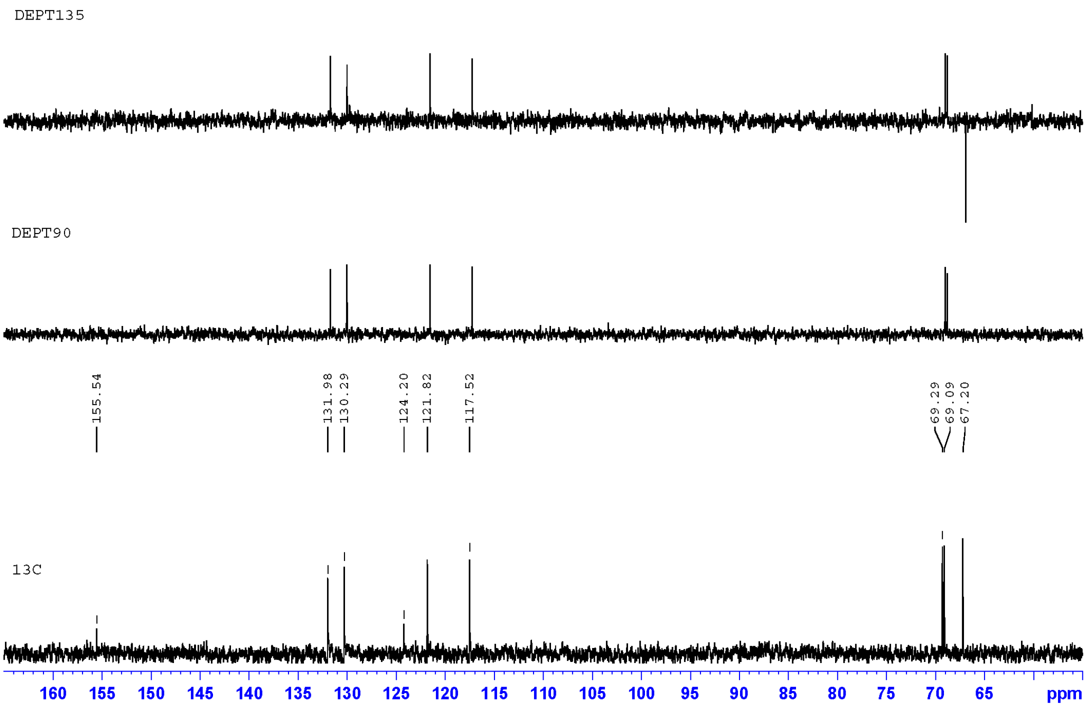

Figure S16. HSQC spectrum of 2-(2-hydroxyphenyl)oxetan-3-ol (**7**).

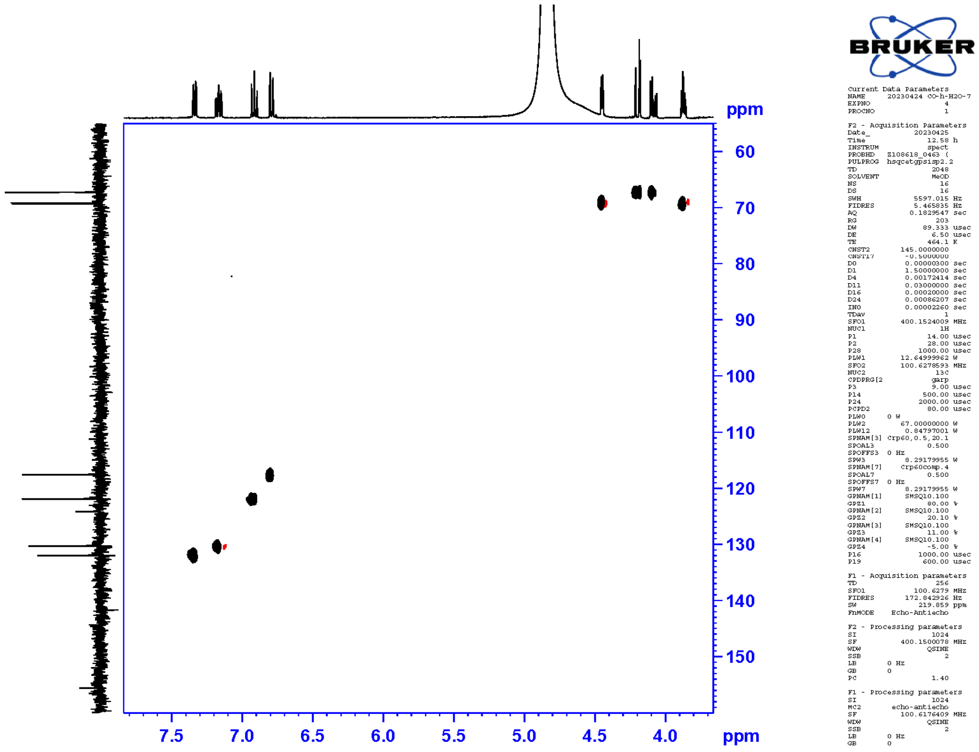

Figure S17. HMBC spectrum of 2-(2-hydroxyphenyl)oxetan-3-ol (**7**).

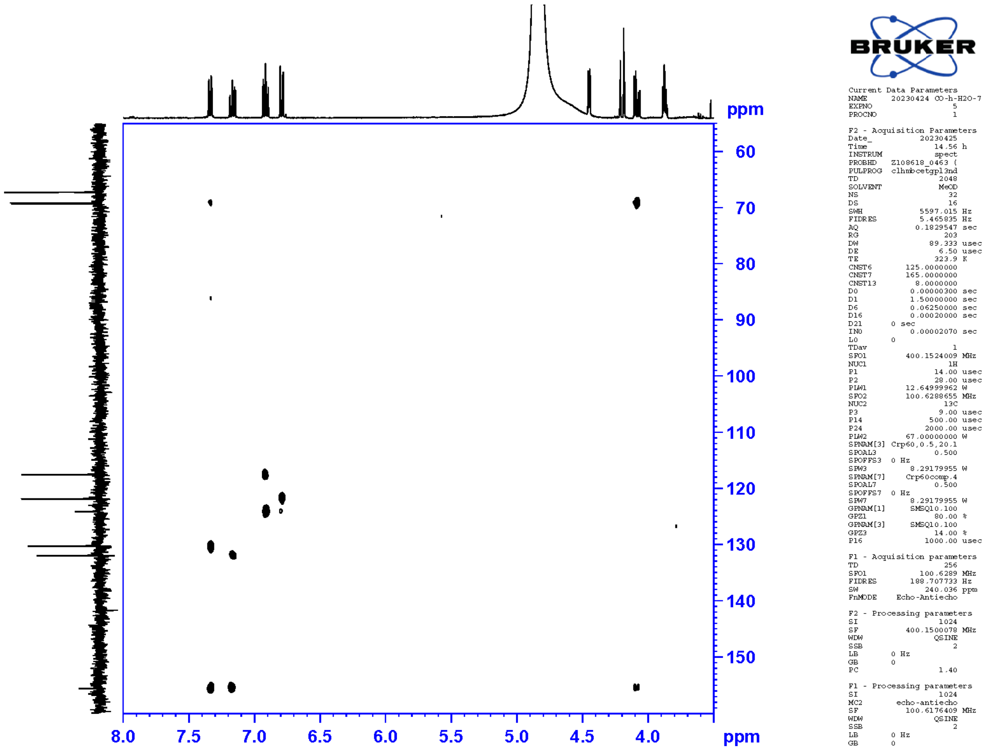

Figure S18. COSY spectrum of 2-(2-hydroxyphenyl)oxetan-3-ol (**7**).

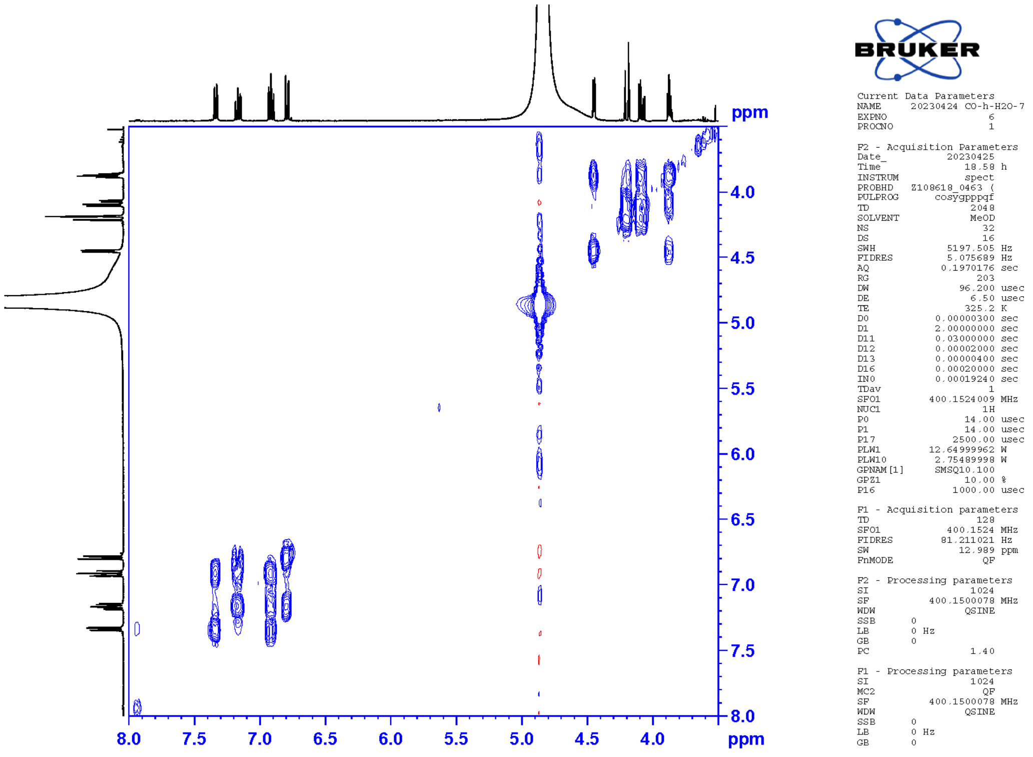

Figure S19. NOESY spectrum of 2-(2-hydroxyphenyl)oxetan-3-ol (**7**).

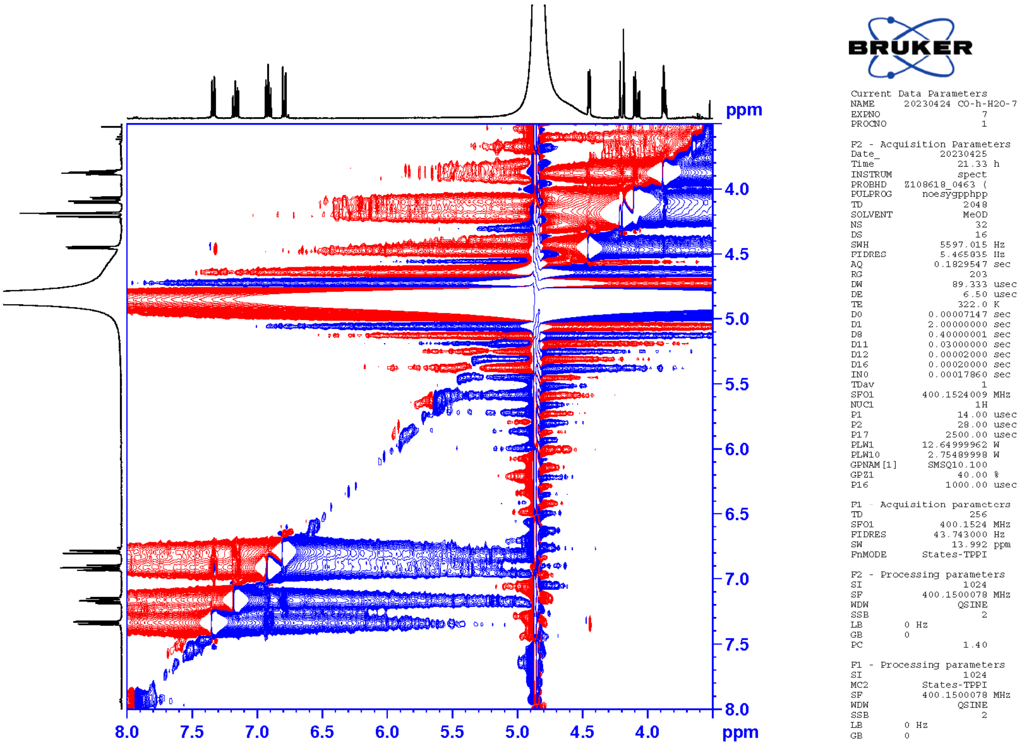

Figure S20. Cytotoxicity of CO hydrosol and its compounds on Hep3B.

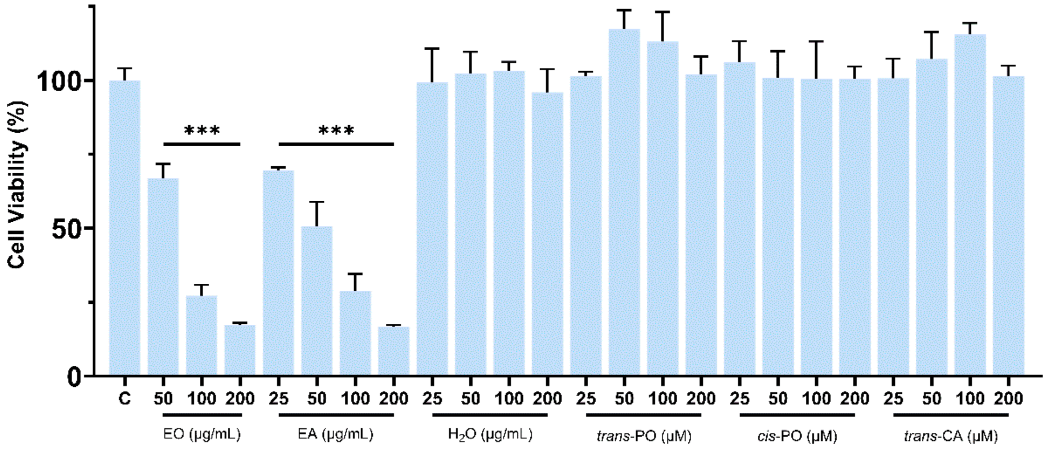

EO: essential oil; EA: hydrosol ethyl acetate fraction; H_2O: hydrosol water fraction; *trans*-PO: *trans*-phenyloxetan-3-ol; *cis*-PO: *cis*-phenyloxetan-3-ol; *trans*-CA: *trans*-cinnamaldehyde.

The Hep3B cells were maintained in Dulbecco's Modified Eagle medium (DMEM; HyClone,Washington, DC, US), supplemented with 10% fetal bovine sera(FBS; Gibco,Waltham, MA, US), 1 mM sodium pyruvate (Corning), and 1%

penicillin/streptomycin (Corning) at 37 °C in a humidified atmosphere of 5% CO_2. Cell viability was measured using a colorimetric MTT assay. Cells were seeded in 96-well plates at a density of 1×10^4 cells/well. Stock solutions of samples in dimethyl sulfoxide (DMSO; Sigma-Aldrich) were stored in the dark at 4 °C. Appropriate dilutions were prepared on the day of the experiments. The final concentration of DMSO did not exceed 0.25% (v/v). After 24 h incubated with samples, the culture medium was removed and replaced with fresh medium containing 0.5 mg/mL of MTT (Sigma-Aldrich) for 1 h. The supernatant was removed and added 100 μL DMSO to dissolve formazan. Absorption at 570 nm for each well was measured using an ELISA microplate reader (BioTeck Instruments, Winooski, VT, USA).
